# Performance of Air-Conditioning System with Different Nanoparticle Composition Ratio of Hybrid Nanolubricant

**DOI:** 10.3390/mi13111871

**Published:** 2022-10-30

**Authors:** Nurul Nadia Mohd Zawawi, Wan Hamzah Azmi, Mohd Fairusham Ghazali, Hafiz Muhammad Ali

**Affiliations:** 1 Center for Research in Advanced Fluid and Processes, Universiti Malaysia Pahang, Lebuhraya Tun Razak, Kuantan 26300, Malaysia; 2 Faculty of Mechanical and Automotive Engineering Technology, Universiti Malaysia Pahang, Pekan 26600, Malaysia; 3 Mechanical Engineering Department, King Fahd University of Petroleum and Minerals, Dhahran 31261, Saudi Arabia; 4 Interdisciplinary Research Center for Renewable Energy and Power Systems (IRC-REPS), King Fahd University of Petroleum and Minerals, Dhahran 31261, Saudi Arabia

**Keywords:** hybrid nanolubricants, composition ratios, PAG, air-conditioning, refrigeration system

## Abstract

To reduce fuel consumption, the automotive air-conditioning (AAC) system’s coefficient of performance (COP) needs to be improved. The use of a diverse selection of hybrid nanolubricant composition ratios is expected to improve the properties of single nanolubricants, resulting in improved AAC system performance. The goal of this study was to find the best combination of hybrid nanolubricants for the best performance of the AAC system. Al_2_O_3_-SiO_2_/PAG hybrid nanolubricants at 0.06% volume concentrations with various composition ratios (20:80, 40:60, 50:50, 60:40, and 80:20) were investigated. An initial refrigerant charge of up to 155 g and a compressor speed of up to 2100 rpm were used in the experiment. The cooling capacity, compressor work, and COP of the AAC system were measured to determine its efficiency. The COP enhancement and compressor work reduction were recorded up to 16.31% and 18.65% for the 60:40 composition ratio, respectively. The maximum cooling capacity up to 75.84% was recorded for the 80:20 ratio, followed by 60:40. The maximum COP value of 8.81 for 155 g of hybrid nanolubricants was obtained at 900 rpm with a 60:40 composition ratio. Therefore, for optimal performance in the AAC system, a 60:40 composition ratio of the Al_2_O_3_-SiO_2_/PAG nanolubricant combination is strongly recommended.

## 1. Introduction

In today’s society, an air-conditioning system in a vehicle is a necessary and indispensable piece of technology [1] as it is used to provide vehicular thermal comfort for the driver and passenger. The effect of cooling the passenger compartment on the vehicle energy consumption and emissions is highly dependent on the thermal comfort [2] in real conditions. There are a few issues with the current automotive air-conditioning (AAC) system including the fact that it increases fuel consumption while cooling the compartments to the desired level of thermal comfort [3,4]. First, vehicles are in a low-efficiency region when operating with the air-conditioning system during idle conditions for an extended period of time [5]. Consequently, increments in the workload, power, and fuel consumption of the vehicles are required to maintain the operation of the AAC system. The next issue is the increase in harmful gasses emitted by vehicles such as carbon dioxide (CO_x_), hydrocarbons (HC), nitrogen oxides (NO_x_), and carbon monoxide (CO) [6,7] during idling and slow moving traffic. The gases released into the atmosphere will cause air pollution [8] and increase the average temperature on Earth. Therefore, these issues are being addressed in order to improve the current AAC system and reduce fuel consumption while increasing the overall efficiency.

The selection of reliable working fluids has a significant impact on the performance of refrigeration systems in terms of achieving the most optimal system configuration. Thus, proper lubricant and refrigerant selection for AAC systems must be considered [9,10,11,12,13]. Some researchers have experimented using nanoparticles [14,15,16,17,18] dispersed in lubricants known as nanolubricants in refrigeration systems to improve their coefficient of performance (COP) and cooling capacity. The utilization of nanoparticles in conjunction with the base fluids or lubricant mixture results in improved thermal characteristics [19,20] and system performance [21,22]. Ohunakin et al. [23] investigated TiO_2_, SiO_2_, and Al_2_O_3_ nanoparticles suspended in the mineral oil lubricant for application in LPG-powered domestic refrigerators. Increments in COP were observed with TiO_2_ and SiO_2_ nanoparticles up to 2.06%. Senthilkumar and Anderson [24] investigated SiO_2_ nanoparticles dispersed in polyolester (POE) oil for the R410A refrigeration system. They discovered that adding nanoparticles to the lubricant improved the refrigeration system’s performance. The maximum COP value of 1.7, minimum compressor work value at 80 W, and a maximum cooling capacity up to 160 W were observed with a refrigerant mass charge of 40 g with 0.4 g/L SiO_2_ nanolubricants.

The use of refrigerant R134a [25,26], especially in refrigeration systems, has been enhanced by various researchers [27,28,29,30]. Subramani and Prakash [31] studied the effects of nanolubricants on the refrigeration system performance. Al_2_O_3_ nanoparticles were dispersed into MO lubricants with refrigerant R134a. The refrigeration system’s power consumption was reduced by 25% when nanolubricants were used. Kumar and Elansezhian [32] analyzed the refrigeration system operating with refrigerant R134a, PAG lubricants, and Al_2_O_3_/PAG nanolubricants. The nanolubricants at a 0.2% volume concentration showed better performance with 10.32% less energy. Joshi et al. [33] performed an investigation on both R134a and R600a using Al_2_O_3_ nanolubricants dispersed in POE and MO, respectively. The power consumption was reduced up to 25.16% using Al_2_O_3_-POE nanolubricants and was further reduced up 28.7% with the R600a system using Al_2_O_3_-MO nanolubricants.

The literature on the use of nanolubricants to enhance the AAC system performance is limited, focusing primarily on single-component nanolubricants [29]. Despite this, no further research into the performance of AAC systems using hybrid nanolubricants has been undertaken in recent years. The hybrid nanolubricants improved the AAC system’s thermal physical properties and performance, resulting in a higher COP and less work for the compressors [30]. However, the effects of the composition ratio of the hybrid nanolubricants toward the AAC system performance have yet to be reported. Thus, there is a need to study the effects of the composition ratio of hybrid nanolubricants toward the performance of the AAC system. Therefore, the purpose of this research was to explore how the composition ratio of nanolubricants affects the performance of the AAC system. The Al_2_O_3_-SiO_2_/PAG at various composition ratios was investigated for different initial refrigerant charges. The optimal volume concentration of Al_2_O_3_-SiO_2_/PAG for use in an AAC system was pursued. The optimum composition ratio of hybrid nanolubricants needs to be investigated by considering the effects of hybrid nanolubricants toward the overall performance of the AAC system.

## 2. Methodology

### 2.1. Preparation of Al_2_O_3_-SiO_2_/PAG Nanolubricants

The Al_2_O_3_ and SiO_2_ metal oxide nanolubricants were prepared in this study using a two-step method [34,35,36] with PAG 46 as the base lubricant. Table 1 describes the characteristics of nanoparticles [37], whilst Table 2 depicts the characteristics of PAG 46 lubricants at atmospheric pressure [34]. The DENSO ND-oil 8, which corresponds to the ISO 46 viscosity class, is approved for the R134a refrigerant [38]. Equation (1) is used to calculate the concentration of nanolubricants in each volume.

(1) $$\phi =\frac{m_{p}/\rho_{p}}{m_{p}/\rho_{p}+m_{L}/\rho_{L}}\times 100$$

where ϕ is the volume concentration of nanolubricants (%); $m_{p}\;$ is the nanoparticle mass (g); $\rho_{p}$ is the nanoparticle density (kg/m^3^); $m_{L}$ is the lubricant mass (g); and $\rho_{L}$ is the lubricant density (kg/m^3^). Nanoparticles added at volume concentrations of more than 0.06% greatly enhanced the coefficient of friction and wear rate, even to values higher than pure PAG [34]. Thus, a maximum volume concentration of 0.06% for various composition ratios was studied.

**Figure 1:**
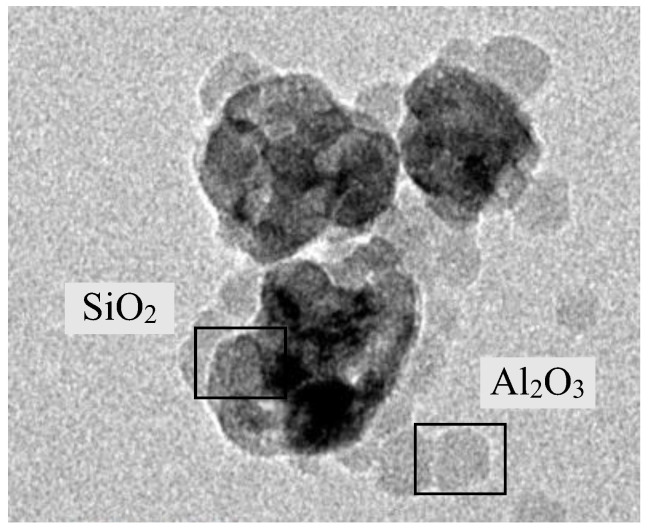
TEM image of the hybrid nanolubricants.

**Figure 2:**
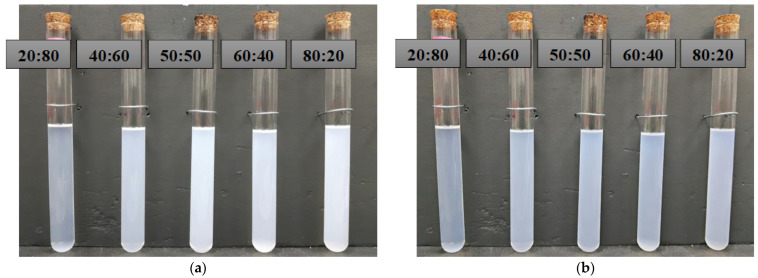
Samples of the Al_2_O_3_-SiO_2_/PAG hybrid nanolubricant after 30 days: (**a**) After preparation; (**b**) after 30 days of preparation.

For a volume concentration of 0.06%, the Al_2_O_3_-SiO_2_/PAG was prepared using a two-step preparation method. The composition ratios of the hybrid nanolubricants investigated in the current study were 20:80, 40:60, 50:50, 60:40, and 80:20, which were determined using the volume percentages of the mono nanolubricants. The Al_2_O_3_-SiO_2_ hybrid nanolubricant composition ratios were prepared in volume percentages in accordance with Table 3, which outlines the composition ratios used in the current study. In preparing Al_2_O_3_-SiO_2_/PAG with a 50:50 ratio, 52.5 mL of Al_2_O_3_/PAG nanolubricant was mixed into 52.5 mL of the SiO_2_/PAG nanolubricant. The nanolubricants were then combined for up to 30 min using a magnetic stirrer before being sonicated for up to two hours. The Al_2_O_3_-SiO_2_/PAG had an optimum stability at a sonication time of 2 h [39,40]. The dispersion of the hybrid nanolubricants was observed using TEM, as shown in Figure 1. After preparation, the dispersion stability of the hybrid nanolubricants was visually examined as well as thirty days later. As seen in Figure 2, no sedimentation appeared in the samples. The average absolute zeta potential reading for a 0.1% volume concentration of Al_2_O_3_-SiO_2_/PAG was up to 61.1 mV. The zeta potential for Al_2_O_3_-SiO_2_/PAG was found to be above the stable limit, suggesting an outstanding stability condition. The sonication duration, stability, and thermal physical features of Al_2_O_3_-SiO_2_/PAG have all been confirmed and verified in previous studies [39].

### 2.2. Automotive Air-Conditioning System Testing Equipment

**Figure 3:**
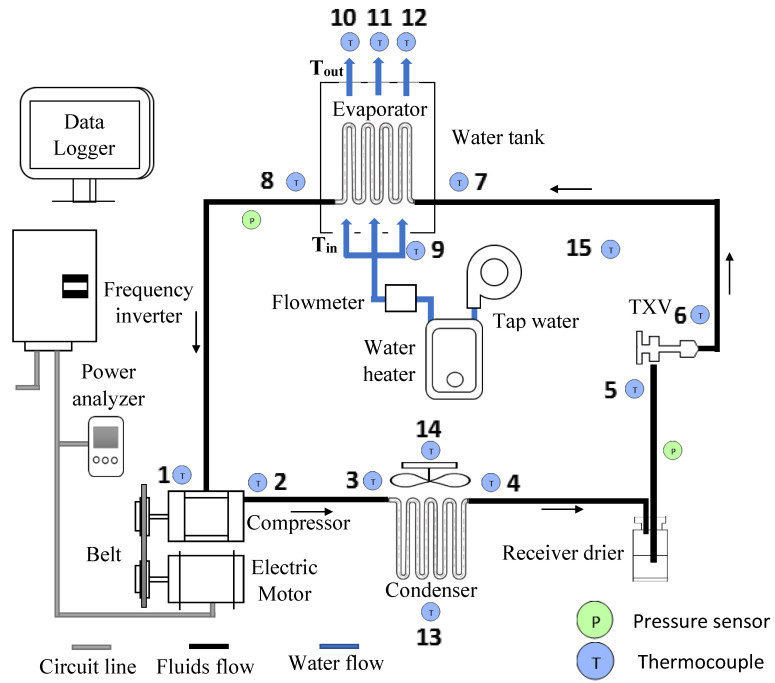
Schematic diagram of the AAC system.

**Figure 4:**
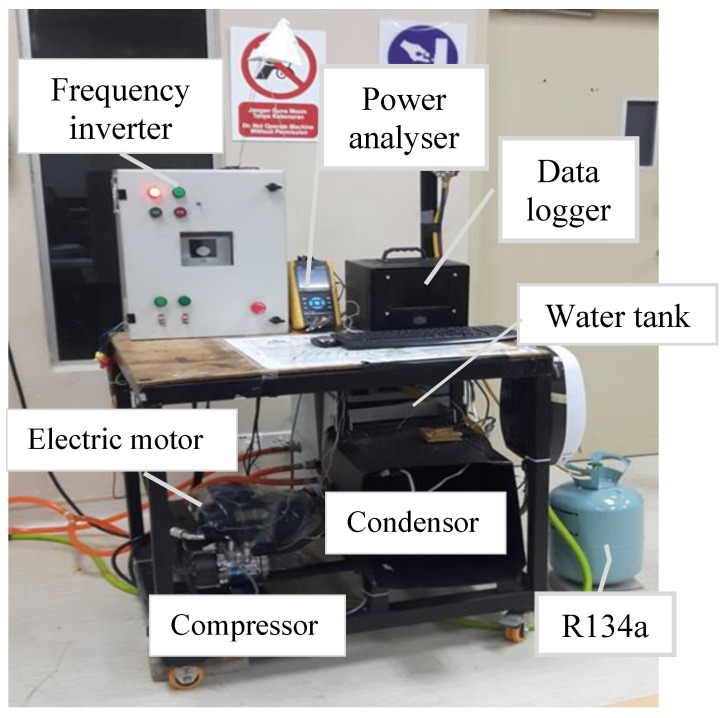
AAC system experimental test bench.

The experimental configuration consisted of the original components of the compact car’s AAC system. The driver, with its control system, the water bath system for the evaporator, the piping system, and other measurement equipment, comprised the bench configuration of the refrigeration system. The compressor, condenser, evaporator, expansion valve, and receiver drier are the essential components of the AAC system. The fixed displacement compressor is commonly used in a compact car type AAC system. The compressor has maximum refrigeration capacities of up to 1.2 kW. The water bath system was a properly insulated 60 liter water tank with a water inlet and outlet for the purpose of the evaporator calorimetric system. The insulated expansion valve and the evaporator were placed inside the tank. The cooling capacity and the mass flow rate of the system were determined using the evaporator calorimetric method in accordance with ASHRAE Standard 41.9 (2000). The inverter frequency controller and the electrical motor were used by the driver with the control system. A 4 kW capacity frequency inverter was employed to run the electrical motor and control the compressor revolution speed. A 2.2 kW 3-phase induction motor drives the compressor via a belting system. The maximum speed of the motor was 2850 rpm. A 3-phase power analyzer from Lutron (model DW-6092) was used to measure the electrical power characteristics and power consumption of the induction motor to drive the compressor. The instrumentation was controlled by a datalogger, which included flow rate sensors, thermocouples, and pressure sensors. The datalogger consisted of a datalogger integrated with 16 channels of the temperature measurements. The 16 channels datalogger represent 16 thermocouple points of measurement as shown in Figure 3. All of the readings were stored automatically in the datalogger. Figure 3 depicts the schematic design of the AAC system bench and Figure 4 shows the AAC system configurations. The Sanden SWJ-7B08 compressor runs on the R134a refrigerant and is lubricated using PAG lubricants. The nanolubricants lubricate the compressor bearings and other moving parts and are carried through the compressor by the refrigerant, where they circulate with the refrigerant throughout the entire system.

**Figure 5:**
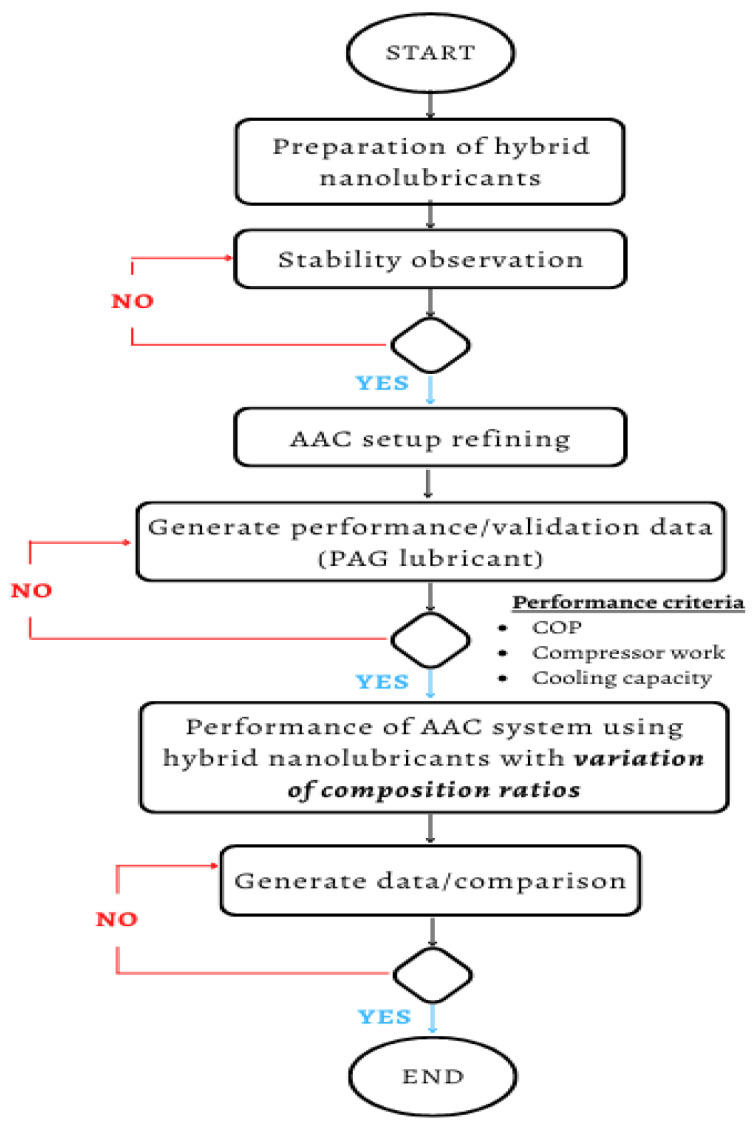
AAC system process flow chart.

Prior to the AAC system trials, the thermocouple, pressure gauge, and flow meter were calibrated in the current investigation. Calibration of the thermocouple, pressure gauge, and flow meter were performed in the present study prior to the AAC system experiments. In this experiment, a K-type thermocouple with a diameter of 0.3 mm and a temperature range of −40 to 375 °C with a tolerance of 1.5 °C was used to monitor the temperature. This was calibrated using the Standard Platinum Resistance Thermometer (SPRT). A K-type thermocouple was chosen because it can tolerate a large temperature range (0 and 1260 °C) and has great oxidation and acidity tolerance due to its material type (chromel and alumel). Similar sensor calibrations were also performed by previous researchers [29,41,42]. The thermocouples (T_1_–T_8_) were used to measure the entering and exiting temperature of the refrigerants for each of the four main components of the AAC system. In addition, the thermocouple monitored the temperature of the inlet (T_in_) and outlet (T_out_) of the water bath and the ambient temperature (T_amb_). Table 4 summarizes the list of sensors and equipment with their uncertainty values. The overall flow chart of the AAC system test run using hybrid nanolubricants with different composition ratios is shown in Figure 5.

### 2.3. Experimental Procedure

The AAC system was flushed with nitrogen (N_2_) gas to remove pollutants and hazardous compounds while also preventing component degradation. The system was then subjected to a leakage test by attaching a vacuum pump to it. Both the flushing and leakage test were performed each time prior to the AAC system experiments. Throughout the experiment, the atmospheric temperature and humidity were measured and regulated between 45.5 and 55.5 °C and 50 and 60%, respectively. It should be noted that the test rig was designed in compliance with the SAEJ2765 (2008) specification and was developed in a control room. The PAG lubricants in a volume of 105 mL were introduced into the AAC compressor for the benchmark study of the AAC system running with PAG lubricants. The system was then put through a leakage test to ensure that no leaks occurred. Following that, 95 g of the R134a refrigerant was added to the system, which charged the system. Water was then pumped into the bath tank until the evaporator was completely immersed. The water heater regulated the water bath’s intake (T_in_) and outlet (T_out_) temperatures until the water flow temperature was uniform. At all times, the inflow flow rate was fixed at 8 liters per minute (lpm). The temperature of the inflow as well as outflow water baths was generally maintained within an allowed range of 30 to 31 °C.

Once all the previous steps had been accomplished, the motor was started, and the compressor’s magnetic clutch was engaged. The frequency meter was adjusted to obtain the required compressor speed of 900 rpm. The datalogger and power analyzer were both turned on at the same time in order to measure the temperature, pressure, power usage, and water mass flow rate. The AAC device was left running for 20 min (to achieve a steady-state condition), while the experimental data were recorded at the same time. The datalogger and power analyzer were turned off at the end of the 20-min experiment period to stop the data collection. The heater, motor, and compressor were all shut off afterward. The system was then run for a second cycle, with the compressor speeds gradually increased up to 2100 rpm and variable initial refrigerant charges of up to 155 g were added. The AAC system was then flushed out before additional experimental testing could begin. Afterward, all of the procedures were duplicated using Al_2_O_3_-SiO_2_/PAG. The experiment was carried out at a constant compressor speed to study the efficiency of the AAC system with various Al_2_O_3_-SiO_2_/PAG composition ratios. The AAC system was then tested with different compressor rates of speed and initial refrigerant loads to find the best composition ratio of Al_2_O_3_-SiO_2_/PAG.

### 2.4. Experimental Analysis

The temperatures at each position in Figure 3 as well as the minimum and maximum pressures (P_low_ and P_high_), which were all recorded in the datalogger, were all obtained to assess the performance of the AAC system. Using information gathered from the datalogger, the performance of the AAC system’s parameters including the cooling capacity, compressor work, COP, and AAC power consumption, was analyzed. Equations (2)–(5) were used to evaluate key parameters such as cooling capacity, compressor work, COP, and AAC power consumption. The enthalpy difference between the water inlet, T_in_, and outlet, T_out_, was used to calculate the cooling capacity ($\dot{Q_{L}}$) of the AAC system. Equation (2) yields the cooling capacity, while Equation (3) calculates the heat absorbed ($Q_{L}$). The heat capacity of water, $C_{p,w}$, was held constant at 4.2 J while its mass flow rate ($\dot{m_{w}}$) was kept at 8 lpm. Equation (4) can be used to obtain the compressor work ($W_{in}$) of the system. Equation (5) can be used to determine the COP of the AAC system. The ratio of heat absorbed to the work undertaken by the compressor to remove the heat is known as the COP. By determining the value of pressure and temperature from the experimental data, the enthalpy of the AAC system is computed. The obtained results were used in conjunction with the properties tables [43] and the psychrometric chart in order to formulate an estimate for the enthalpy.

(2) $${\dot{Q}}_{L}={\dot{Q}}_{water}={\dot{m}}_{w}c_{p}{}_{,water}(\Delta T)$$

(3) $$Q_{L}=h_{1}-h_{6}$$

(4) $$W_{in}=h_{2}-h_{1}$$

(5) $$\mathrm{COP}=\frac{Q_{L}}{W_{in}}=\frac{h_{1}-h_{6}}{h_{2}-h_{1}}$$

## 3. Results and Discussion

### 3.1. AAC Performance Using PAG Lubricants

**Figure 6:**
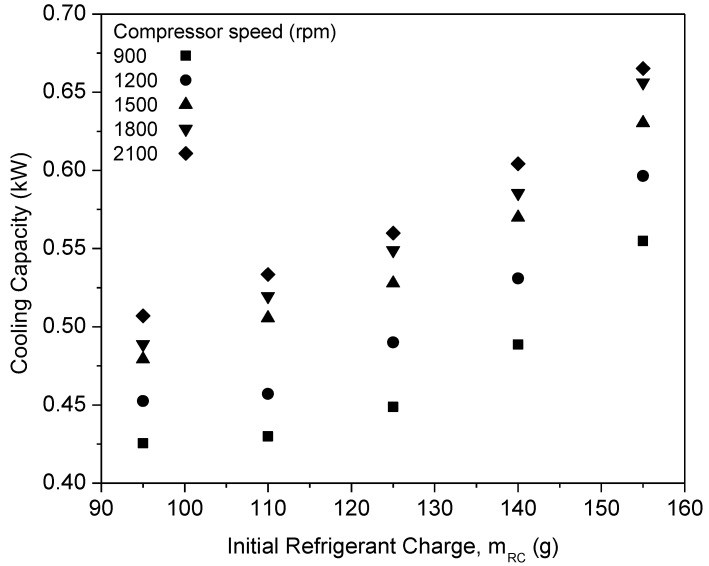
Cooling capacity of pure PAG at various refrigerant charges and compressor speeds.

The refrigerant charge is a crucial aspect in the evaluation of the refrigeration system performance. This is a result of insufficient refrigerant charge reducing the COP and increasing the energy consumption [44]. Thus, the refrigerant charge will affect the performance of the system. This is due to the increase in compressor work along with the increase in the refrigerant charge amount. It is important to study the optimal charge of the refrigeration system to optimize the performance and efficiency of the refrigeration system. Consequently, the refrigerant charge will influence the performance of the system. This is due to the increase in compressor work and the rise in the amount of refrigerant charge. It is essential to examine the appropriate charge of the refrigeration system in order to enhance its performance and efficiency. Following a setup evaluation of the AAC system, the AAC system studies were commenced. The AAC system performance, specifically cooling capacity, coefficient of performance (COP), and compressor work were studied using a range of R134a refrigerant charges from 95 to 155 g. The performance of pure PAG lubricants employed in the AAC system acted as the reference data for the relative performance validation of hybrid nanolubricants. The cooling capability of pure PAG lubricants at various refrigerant loads is shown in Figure 6. The cooling capacity rose as the starting refrigerant charge and speed increased, as seen in the graph. The conservation of mass equation was demonstrated by the cooling capacity increasing as the mass flow rate increased. The mass flow rate tended to rise with an increasing compressor speed, thus increasing the cooling capacity [29]. The compressor speed increment will increase the system’s high side pressure, thus increasing the enthalpy of the saturated refrigerant. Additionally, when the system’s mass flow rate increases, the evaporator’s temperature rises [45]. This is due to the fact that when the system’s mass flow rate increases, the evaporator’s temperature rises [45]. Due to the two-phase refrigerant flow from the condenser to the expansion valve, a reduced cooling capacity was observed at a lower refrigerant charge [29].

**Figure 7:**
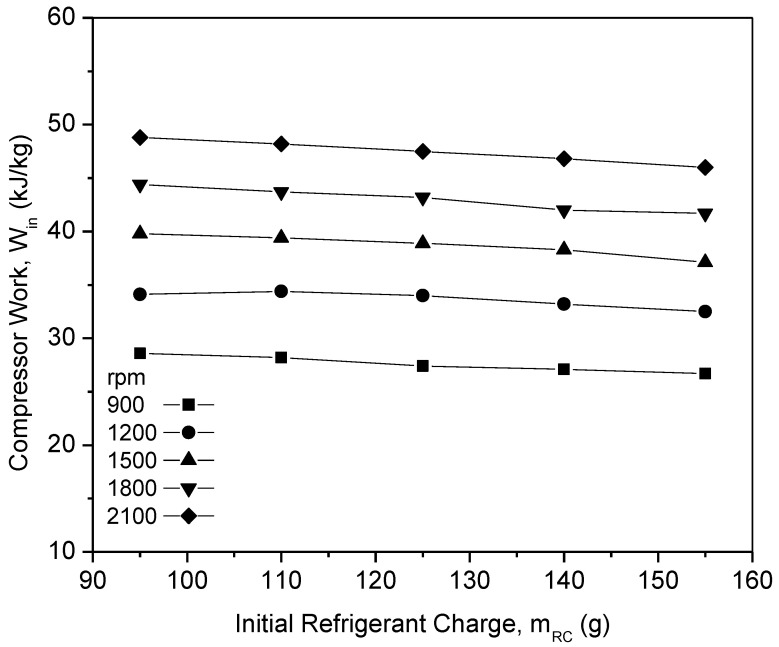
Compressor work of PAG at various refrigerant charges and compressor speeds.

**Figure 8:**
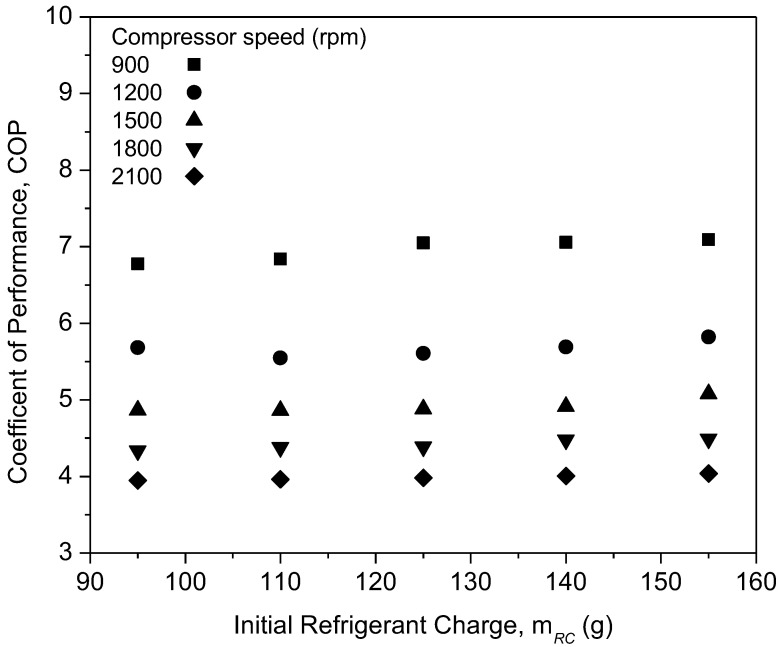
Coefficient of performance (COP) of the pure PAG at various refrigerant charges and compressor speeds.

Figure 7 shows the graph of the compressor work of PAG against refrigerant charges with a variation in the compressor speeds. From the graph, the compressor work of pure PAG lubricants showed a small decrease in the compressor work with an increase in the initial refrigerant mass. However, the compressor work increased with the increase in speed. This was due to increments in the discharge temperature and pressure at the compressor along with the increasing compressor speed. Therefore, increasing the enthalpy difference will result in an increment in the compressor work. When operating at higher speeds, compressors typically exert a much greater amount of effort than when operating at lower speeds. Thus, this will also lead to a decrease in the efficiency of the system. A similar trend was observed for the compressor work in the literature [46,47]. Figure 8 shows the graph of the COP for the PAG based lubricants against the initial refrigerant charge. The most commonly analyzed main parameter for measuring the air-conditioning system performance is the COP [48]. With an increasing refrigerant mass and compressor speed, the system COP increases considerably. This is because of the increase in the cooling capacity, which overweighs the increment of the compressor power at the higher compressor speed and refrigerant charge. At a compressor speed of 900 rpm and a refrigerant load of 155 g, the COP reached its maximum of 7.09. However, due to limitations in the AAC setup and to avoid compressor operation overload, the AAC system was charged up to 155 g only. Overload compressor operation can lead to performance degradation within the compressor [49]. Hence, Al_2_O_3_-SiO_2_/PAG hybrid nanolubricants in this study were evaluated up to 155 g of refrigerant charge of the AAC system performance for further investigations.

### 3.2. AAC Performance Using Al_2_O_3_-SiO_2_/PAG Hybrid Nanolubricants

#### 3.2.1. Nanolubricants with Composition Ratios

**Figure 9:**
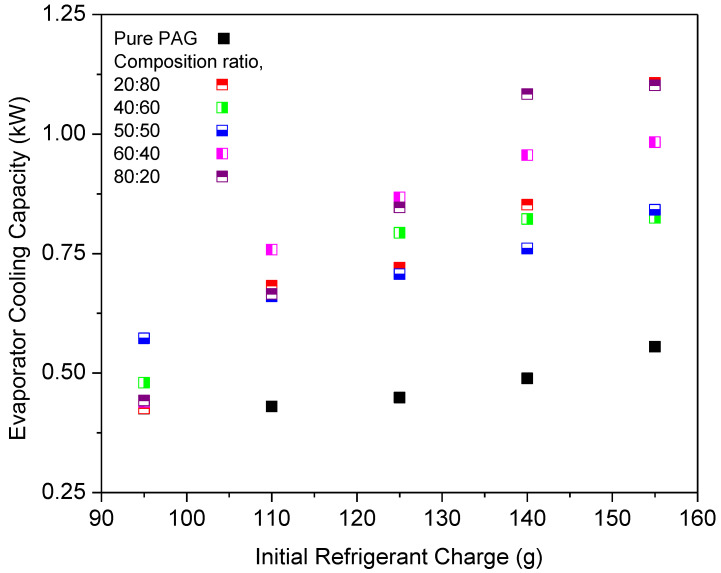
Cooling capacity of hybrid nanolubricants at various composition ratios.

Figure 9 depicts the cooling capacity of various composition ratios of Al_2_O_3_-SiO_2_/PAG against the initial refrigerant charge at 900 rpm. The cooling capacity of hybrid nanolubricants increased along with the increase in the initial refrigerant charge. It was discovered that Al_2_O_3_-SiO_2_/PAG lubricants had a better cooling capability than the pure PAG lubricants. This is because the nanoparticles in hybrid nanolubricants help to improve heat transmission, causing the heat transfer in the evaporator to increase. Moreover, with the addition of nanoparticles in the lubricants, the decrement in the heat transfer coefficient can be avoided in the evaporator [50]. The Al_2_O_3_-SiO_2_/PAG hybrid nanolubricants have better thermal physical properties than the PAG lubricant [51]. The nanoparticles in hybrid nanolubricants improve heat transmission, resulting in an increase in heat transfer in the evaporator. Consequently, there is a correlation between the thermal conductivity and the composition ratios of the hybrid nanolubricants, and cooling capacity enhancements can be attributed to this correlation. The highest cooling capacity enhancement was achieved for the 80:20 ratio with 75.84% improvement for the hybrid nanolubricants compared to the pure PAG lubricants. The cooling capacity at the 60:40 ratio was enhanced up to 70.94% and higher than the 20:80 ratio. However, the 50:50 ratio showed an improvement of 45.40% and found the lowest cooling capacity enhancement. The composition ratio with more than 50% Al_2_O_3_ nanoparticles contributed to high cooling capacity enhancements up to 70%.

**Figure 10:**
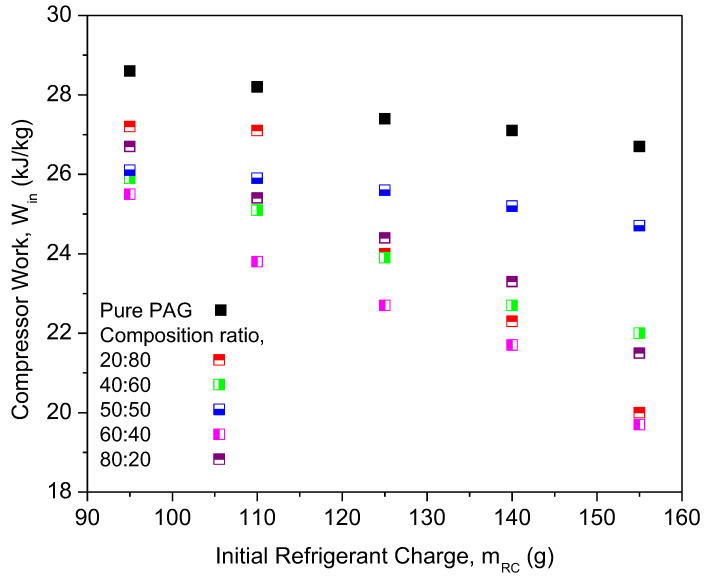
Compressor work of hybrid nanolubricants at various composition ratios.

**Figure 11:**
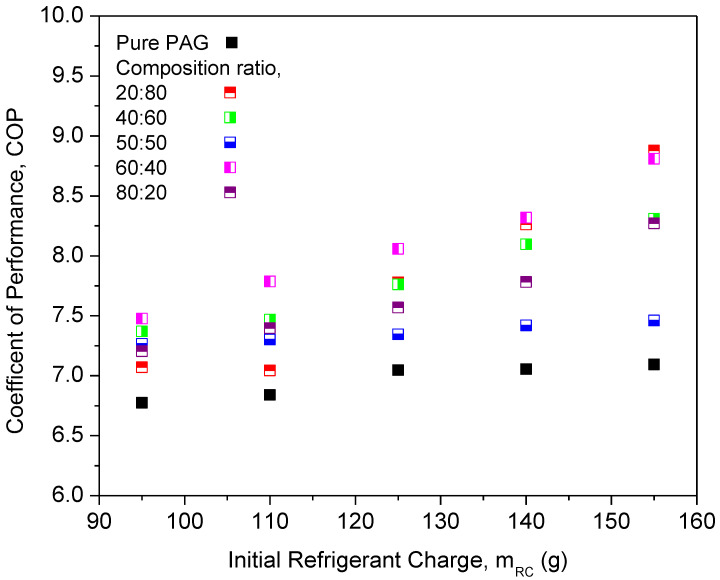
Coefficient of performance of hybrid nanolubricants at various composition ratios.

The compressor work of different composition ratios was plotted against the initial refrigerant mass, as shown in Figure 10. The highest compressor work was noticed for the PAG lubricants. This indicates that the compressor using pure PAG lubricants requires more work compared to the nanolubricants under similar working conditions. The compressor work using hybrid nanolubricants at the 60:40 ratio exhibited the highest reduction of 17.95% and the lowest reduction of 7.59% at a ratio of 50:50. The composition ratios of hybrid nanolubricants can greatly influence the compressor work of the AAC system. Due to the optimal composition ratios of hybrid nanolubricants, the compressor workload is minimized as the friction coefficient decreases. Therefore, reducing the friction coefficient is crucial to cutting the energy consumption of the compressor. However, the 50:50 ratio can only slightly reduce the compressor work when compared with other ratios due to high viscosity, especially for high refrigerant charges. Less work due to lower viscosity gives an advantage to the compressor operation. Viscosity of the lubricants is one of the major factors for compressor performance in the refrigeration system [37]. From Figure 11, the COP of Al_2_O_3_-SiO_2_/PAG with different composition ratios increased with a refrigerant charge. All composition ratios showed enhancements in COP performance compared to the pure PAG. The COP performance for the 60:40 ratio exhibited the highest enhancement of up to 16.31%. Meanwhile, the lowest COP enhancement of 5.7% was attained for the 50:50 ratio. A high percentage of SiO_2_ nanoparticles in the hybrid nanolubricants greatly contributed to the COP enhancement. Meanwhile, a high percentage of Al_2_O_3_ nanoparticles can lower COP enhancement. The 60:40 ratio exhibited better overall performance in terms of cooling capacity, compressor work, and COP. The average enhancement and reduction in the AAC system for all composition ratios are summarized in Table 5. In this study, the experimental setup was used to identify the optimal hybrid nanolubricant design for the AAC system performance. The optimization analysis process can then be further validated using any available techniques such as the Taguchi method, response surface technology methods (RSM) [40], machine learning (ML) based intelligent techniques, meta-heuristic algorithms [52], and other optimization techniques using the experimental data that were obtained.

#### 3.2.2. Nanolubricants with Compressor Speeds

**Figure 12:**
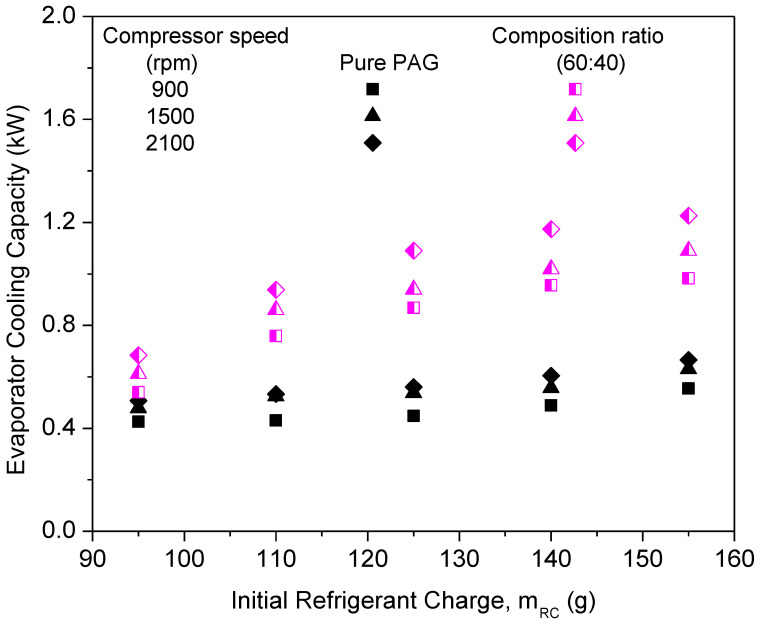
Cooling capacity of the hybrid nanolubricants at the optimum 60:40 composition ratio.

**Figure 13:**
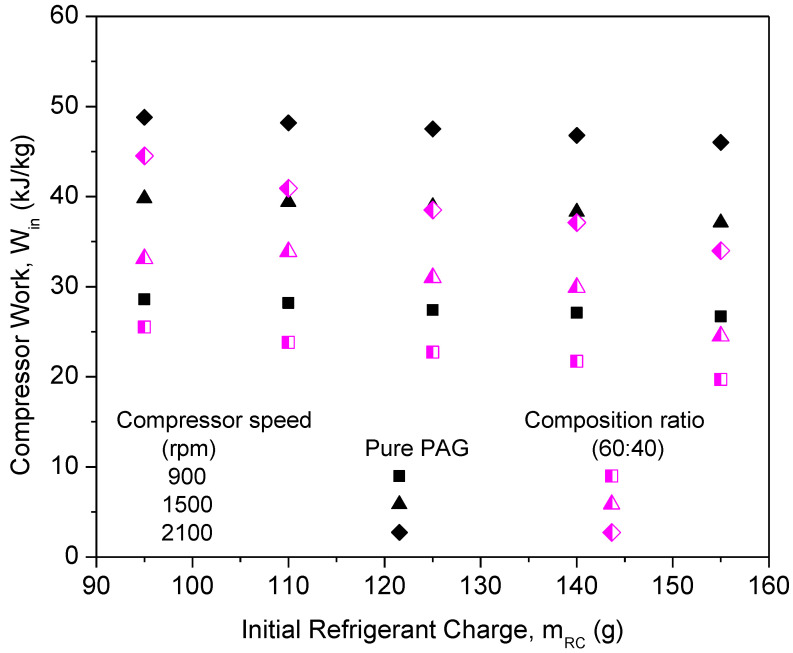
Compressor work of hybrid nanolubricants at the optimum 60:40 composition ratio.

**Figure 14:**
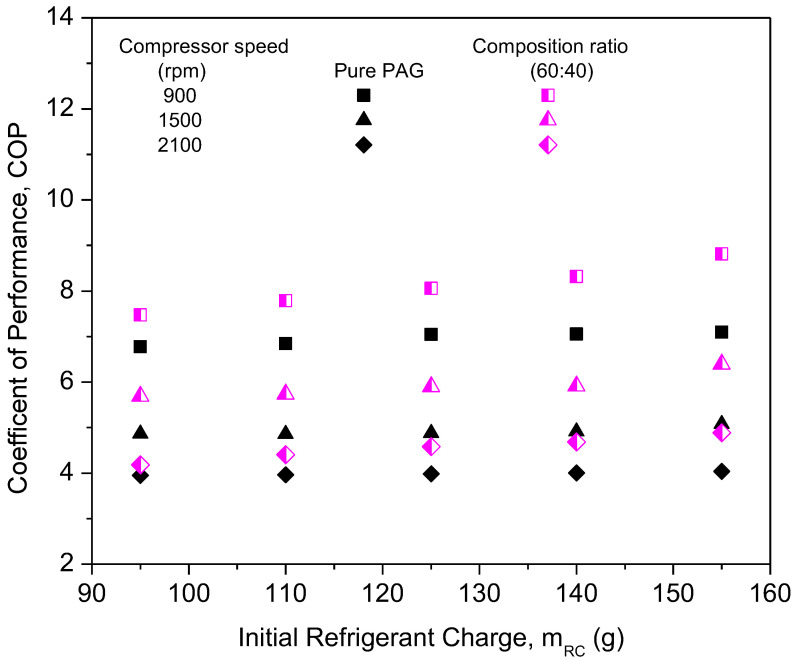
Coefficient of performance of the hybrid nanolubricants at the optimum 60:40 composition ratio.

The AAC system performance, namely cooling capacity, compressor work, and COP were investigated for various compressor speeds at a 0.06% volume concentration and 60:40 composition ratio of Al_2_O_3_-SiO_2_/PAG hybrid nanolubricants. The performance of the AAC system was investigated at different compressor speeds varying from 900 to 2100 rpm. Figure 12, Figure 13 and Figure 14 indicate the performance parameters of the Al_2_O_3_-SiO_2_/PAG composite nanolubricants at different compressor speeds as a function of the initial refrigerant charge. Figure 12 shows the cooling capacity for hybrid nanolubricants with 60:40 at different compressor speeds. As the compressor speed rises, the system’s cooling capacity also rises. The Al_2_O_3_-SiO_2_/PAG lubricant had a significantly larger cooling capacity than the pure PAG lubricants at all compressor speeds. The incorporation of nanoparticles to PAG lubricants increased the system’s cooling capability. A system with a large cooling capacity has superior heat transfer performance and can cool down the system more effectively. Enhancement in the thermal conductivity of nanolubricants improves the heat absorbance and utilizes the system to operate at the utmost efficiency. The improvement in the cooling capacity is in line with the thermal conductivity evaluation previously reported [39,53].

Figure 13 represents the effects of compressor work at different compressor speeds for Al_2_O_3_-SiO_2_/PAG at the 60:40 ratio. When compared to the pure PAG lubricants, the compressor work for hybrid nanolubricants was significantly less. In addition, when the refrigerant charge rises, the compressor’s workload diminishes. Low compressor speed is thought to result in less load on the compressor, resulting in less compressor effort compared to greater compressor speed. When compared to the pure PAG, Al_2_O_3_-SiO_2_/PAG contributes more to lowering the compressor effort. This is because of a decrease in the tribological behavior of hybrid nanolubricants, specifically the friction coefficient and the percentage of wear. Thus, in order to reduce the compressor friction losses, which lower the pressure drop and compressor effort, hybrid nanolubricants are required in the refrigeration systems. The hybrid nanolubricants are being considered since a higher coefficient friction results in greater friction losses. Figure 14 shows a graph of COP against the initial refrigerant mass of the system at different compressor speeds for the 60:40 ratio. The graph indicates that COP improves as the refrigerant charge increases, but declines as the compressor speed increases. The graph depicts a low COP for the pure PAG lubricants. This is because the pure PAG lubricants have low cooling capacities but high compressor work. The maximum COP achievable was 8.81 for Al_2_O_3_-SiO_2_/PAG of the 60:40 ratios at the 155 g refrigerant charge and 900 rpm. Sabareesh et al. [54] and Kumar and Elansezhian [32] both found that this pattern persisted. Al_2_O_3_-SiO_2_/PAG was observed to have a similar pattern in COP increments with the pure PAG lubricants. High compressor speeds result in more work being done by the compressor, resulting in a decrease in COP. In order to overcome this, the compressor’s workload must be reduced. Consequently, the efficiency of the compressor is improved.

## 4. Conclusions

The performance of the AAC system was tested at various composition ratios, compressor speeds, and initial refrigerant charges. The cooling capacity, compressor work, and COP were plotted and discussed in relation to the initial refrigerant charges. The optimum AAC system performance using Al_2_O_3_-SiO_2_/PAG was investigated using hybrid nanolubricants with various composition ratios to achieve the optimum AAC system performance efficiency. The COP is highlighted as the main priority to determine the optimum composition ratio of nanolubricants for the performance of the AAC system. The highest average enhancement of 16.31% for COP was recorded with the 60:40 ratio at constant volume concentration. The lowest average enhancement of 5.70% was at the 50:50 ratio. Al_2_O_3_-SiO_2_/PAG exhibited the highest COP value compared to other composition ratios. The effectiveness of the system can be improved by using nanolubricants with the appropriate composition ratio, which has a greater potential for enhancing the COP performance, automotive cooling rates, and a reduction in the compressor work. Due to their remarkable improvement in thermal, physical, and heat transfer capabilities to increase the efficiency and dependability of the refrigeration and air conditioning systems, hybrid nanolubricants have additional advantages over conventional fluids and single-component nanolubricants. Therefore, for the best performance in refrigeration systems, Al_2_O_3_-SiO_2_/PAG hybrid nanolubricants with a 60:40 ratio are advised. Performance optimization of Al_2_O_3_-SiO_2_/PAG refrigeration systems is required to validate the existing findings. The current study was a short-term laboratory experiment that ruled out the AAC system durability using hybrid nanolubricants. To extend the present work, additional research is needed to optimize the performance parameters and durability effects of Al_2_O_3_-SiO_2_/PAG composite nanolubricants on the performance and application of an AAC system.

## Figures and Tables

**Table 1:** Properties of the nanoparticles [37].

Properties	Al_2_O_3_	SiO_2_
Molecular mass (g/mol)	101.96	60.08
Average particle diameter (nm)	13	30
Density (kg/m^3^)	4000	2220
Thermal conductivity (W/m·K)	36	1.4
Specific heat (J/kg·K)	773	745

**Table 2:** Properties of the PAG 46 lubricant at atmospheric pressure [34].

Properties	PAG 46
Density, g/cm^3^ @ 20 °C	0.9954
Flash Point, °C	174
Kinematic viscosity, cSt @ 40 °C	41.4–50.6
Pour Point, °C	−51

**Table 3:** Designated nanoparticle composition ratios.

No.	Composition Ratios of Hybrid Nanolubricants (ɸ %)	
	Al_2_O_3_	SiO_2_
1	20	80
2	40	60
3	50	50
4	60	40
5	80	20

**Table 4:** Uncertainties of the AAC system sensors and measurement devices.

Sensors/Devices	Range	Uncertainty
Tachometer (rpm)	0–20,000	±2.0
Thermocouple (°C)	−40–375	±1.5
Pressure gauge (kPa)	0–2500	±0.5
Water flow meter (LPM)	0–100	±1.0
Weighing scale (kg)	0–25	±0.001

**Table 5:** Average enhancement for AAC system performance with variations in the Al_2_O_3_-SiO_2_ composition ratios.

Composition Ratio of Al_2_O_3_-SiO_2_/PAG	Average Enhancement (%)		Average Reduction (%)
	Cooling Capacity (kW)	COP	Compressor Work (kJ/kg)
20:80	60.19	15.77	16.13
40:60	51.10	12.02	13.62
50:50	45.40	5.70	7.51
60:40	70.94	16.31	18.65
80:20	75.84	7.56	10.87

## Data Availability

Not applicable.
